# The State of Higher Education in Occupational Health and Safety in Central Asian Countries

**DOI:** 10.29024/aogh.2322

**Published:** 2018-08-31

**Authors:** Kenesh O. Dzhusupov, Cholpon T. Sulaimanova, Karlygash K. Toguzbayeva, Ramin Tabibi, Bakhtiyar Serik, Cholpon K. Chonbasheva, Khusseyn Egamnazarov, Maigul S. Kainarbayeva

**Affiliations:** 1 International School of Medicine, Bishkek, KG; 2 Asfendiyarov Kazakh National Medical University, Almaty, KZ; 3 Abadan School of Medical Sciences, Abadan, IR; 4 Karaganda State Medical University, Karaganda, KZ; 5 Akhunbaev Kyrgyz State Medical Academy Bishkek, KG; 6 Avicenna Tajik State Medical University, Dushanbe, TJ

## Abstract

A healthy workforce is vital for the sustainable social and economic development of any country. Assuring occupational health and safety (OHS) depends not only on the passing of quality working legislation and inspection of workplaces, but also on preparation of qualified specialists on OHS.

Aimed at assessing of relevance of the content of training at Central Asian universities to the needs in prevention of risks of accidents and injuries and promotion of a preventive culture in the workplace, and finding out how they are compliant with the recommendations of the Mainstreaming occupational safety and health into the education (2010), we examined curricula of 20 medical and non-medical universities across Kazakhstan, Kyrgyzstan, Tajikistan, Turkmenistan and Uzbekistan.

The analysis of training programs showed that education in OHS and preparation of qualified specialists in Central Asiana countries is up-to-date and tailored to local needs for prevention of risks of accidents and injuries and promotes the notion of a preventive culture in the workplace.

## Introduction

A healthy workforce is vital for sustainable social and economic development of any country. Assuring occupational health and safety (OHS) depends not only on the passing good working legislation and inspection of workplaces but also on preparation of qualified specialists in OHS. In the Global Plan of action on Workers’ Health 2008–2017, the World Health Organization (WHO) urged the governments to strengthen the development of human resources for workers’ health (Objective 3; 18). It should be done by “further postgraduate training in relevant disciplines; building capacity for basic occupational health services; incorporating workers’ health in the training of primary health care practitioners and other professionals needed for occupational health services; creating incentives for attracting and retaining human resources for workers’ health, and encouraging the establishment of networks of services and professional associations. Attention should be given not only to postgraduate but also to basic training for health professionals in various fields such as promotion of workers’ health and the prevention and treatment of workers’ health problems [1].”

The European Agency for Safety and Health at Work indicated challenges to integrating OHS into university-level education compared to other levels of education [2]. In its report it recommended to include OHS as an obligatory element in courses at university level.

The purpose of this work is: 1) to assess the relevance of the training content at Central Asian universities to the needs in preventprevention of risks of accidents and injuries and to the promotion of a preventive culture in the workplace, and 2) how they are compliant with the recommendations of the mainstreaming occupational safety and health into the education (2010).

With this purpose we examined curricula of 20 medical and non-medical universities across five Central Asian countries: Kazakhstan, Kyrgyzstan, Tajikistan, Turkmenistan and Uzbekistan.

## Materials and Methods

For the present study we searched literature sources available in English and Russian languages. We searched electronic databases such as PubMed, Medline, eLibrary, and Google Scholar. The key terms used were: history of education in occupational health, education in occupational health and safety, universities, Central Asia (in course: Kyrgyzstan, Kazakhstan, Uzbekistan, Tajikistan and Turkmenistan). We also manually searched various hard copies of local journals and books in the libraries of medical universities, libraries of the Ministries of Health of Tajikistan, Kyrgyzstan and Kazakhstan, and the Kyrgyz, Tajik and Kazakh national libraries. We also conducted review and analyses of curricula and syllabi on Occupational Health for undergraduate, graduate and postgraduate students, materials of international conferences and round tables, and educational projects.

Included articles would address any aspect of the history and present state of education in occupational health and safety in Central Asian countries such as Kazakhstan, Kyrgyzstan, Tajikistan, Uzbekistan and Turkmenistan. Works with the general description of medical education in these countries were excluded.

In total, we reviewed 19 journal articles in English and 12 journal articles in Russian published at earliest in 1963; two books in English and four books in Russian, one encyclopedia and one textbook in English, one textbook and one national guideline in Russian, and four ILO national profiles in English. Twenty relevant articles and all other publications were considered for the present review.

## Results

The review of literature on the development of occupational health and education on OHS in Central Asian countries showed the long history (Table 1).

**Table 1:** History of the development of Occupational Health in Central Asia.

Author/date	Type of publication	Language	Topic/focus/questions	Time period	Place	Highlights

Malan RM. (1963)	Journal article	Eng	History of OH in Eastern Europe (USSR): OHS services, education and research	19–20 CE	Russia, USSR	The role of A.N. Nikitin and F.Erisman as pioneers of IH and OH
Mohammadali M. et al. (2007)	Journal article	Eng	Review of major influential Persian periods and the individuals who contributed to the development of anatomy	10 BC–14 AD	Persia, Central Asia	direct involvement of Avicenna, Rhazes and other scholars in human dissection
Azizi. M-H. (2007)	Journal article	Eng	Contributions of Avicenna’s “Canon of Medicine” and Rhazes’s “Liber Continens” to the development of otorhinolaryngology	865–1037 AD	Persia, Central Asia	Rhaze’s tracheostomy technique, description of pollynosis Avicenna’s view on anatomy, hygiene, infectious diseases of ENT and its treatment
Modanlou HD. (2008)	Journal article	Eng	Analysis of the chapter of Avicenna’s “Canon of Medicine” about the care of newborns	980–1037 AD	Persia, Central Asia	Description of Avicenna’s view on care of infants, their hygiene (bathing, swaddling, etc), breastfeeding and upbringing
Nayernouri T, Azizi M. (2011)	Journal article	Eng	Analysis of the text of the oldest known medical treatise in Persian language	800–1110 AD	Central Asia	Medicine defined as an art of maintaining a healthy body. The role of diet, hygiene and sport. Clinical description of meningitis, surgical operation of short frenum
Nejabat M, et al. (2012)	Journal article	Eng	Contribution of Avicenna’s “Canon of Medicine” to diagnosis and treatment of eye diseases	980–1037 AD	Central Asia	Avicenna’s classification and diagnostics of cataracts
Zargaran A, et al. (2013)	Journal article	Eng	Focused on of Avicenna’s “Canon of Medicine”, his description of definitions and etiology, diagnostics, clinical manifestations, prognosis of stroke	980–1037 AD	Persia, Central Asia	Concurrency of Avicenna’s definitions and understanding of stroke with current concepts
Nayernouri T. (2015)	Journal article	Eng	History of Iranian Medicine, surgery and pharmacology including input of Avicenna	V BC–1122 AD	Persia, Central Asia	Place of Persian medicine and the role of Avicenna in the history of medicine
Ghaffari F, et al. (2015)	Journal article	Eng	Avicenna’s viewpoint on spinal trauma and its treatment in his “Canon of Medicine”	980–1037 AD	Persia, Central Asia	Description of levels and kinds of spinal impairments caused by spinal traumas; its treatment based on etiology. Usage of different treatment methods (food and drug therapy, massage, phlebotomy, cupping, dry sauna, and surgery).
Buranova DD. 2015	Journal article	Eng	Short biography of Avicenna, his contribution to the development of the world’s medicine	980–1037 AD	Uzbekistan	Avicenna’s view on drug therapy; concurrency of his concepts to the modern medicine
Sadykova JM, et al. (2015)	Journal article	Eng	Needs analysis for retraining and advanced training of specialists in OHS in Kazakhstan	2015	Kazakhstan	Description and assessment of the existing training programs Recommendations to improve training programs
Moradi Z, et al. (2016)	Journal article	Eng	Avicenna’s views on the causes of intestinal obstruction and their comparison to current views	980–1037 AD	Persia, Central Asia	There are some missing etiologies of intestinal obstruction in modern medicine
Dalfardi B, et al. (2017)	Journal article	Eng	Focused on of Avicenna’s “Canon of Medicine”, his description of transmission route, clinical manifestation and treatment of infectious diseases	980–1037 AD	Persia, Central Asia	Detailed description of rabies among a number of infectious diseases. The role of control of insects and rodents, supervision of domestic animals
Goncharenko YuF, Chernobrova OV. (1982)	Journal article	Russian	History of development of the subject “occupational diseases” with stress on Russian and USSR period of developments	1700–1980	Russia, the USSR	The role of Russian doctors, scientists and Russian Emperors in the establishment of scientific school of industrial hygiene and studies of occupational diseases
Voskresenskaia NP, Bylinskiĭ EN. (2003)	Journal article	Russian	The history of preventive medicine with the focus on Russian and USSR developments	1714–2000	the USSR, Russian federation	The role of Petr I and Anna Empress in the development of preventive medicine
Izmerov NF. (2006)	Journal article	Russian	The role of G.V. Khlopin in the development of the Russian and Soviet school of occupational health	1900–1925	Russia, the USSR	Khlopin’s large-scale experimental work on the effect of industrial poisons on the body, the physiology of labor (energy expenditure)
Izmerov NF. (2009)	Journal article	Russian	Role of F.F. Erisman in development of occupational hygiene	1841–1915	Russia	Results of the first big examination of Russian workers
Otarbayeva MB. (2015)	Journal article	Russian	Role of Kazakh National center for industrial hygiene and occupational diseases in training of occupational therapists in Kazakhstan	1950–2015	Kazakhstan	Current issues of preparation of specialists in OHS in Kazakhstan. History of the National Center for IH and OD
Shigan EE. (2016)	Journal article	Russian	Analysis of main works of F. Erisman, their significance	19 CE	Russia	The role of F. Erismann in the development of OH as a science and discipline
Shigan EE. (2016)	Journal article	Russian	Main stages of the development of Preventive medicine, the role of G.V. Khlopin, F. Erisman, A.A. Nikitin and others in history of OHS in USSR	19–20 CE	Russia, the USSR	The role of G.V. Khlopin in OHS history; Main publications of N.A. Nikitin
Avicenna	Book	Eng	Knowledge presented by Canon Basic and special differences between Canon and modern medicine	980–1037 AD	Central Asia	Basic and special differences between Canon and modern medicine
Avicenna (World Digital Library	Book	Eng, Arabic	Anatomy of human body, philosophy of well-being of a man, causes of disease, manifestations, treatment, dietetics, hygiene, environment al factors etc	980–1037 AD	Persia, Central Asia	Role of environmental factors, including working conditions Metal poisonings, their treatment
Gutas D. (2016)	Encyclopedia	Eng	Biography of Avicenna, his contribution to the development of the world’s medicine, philosophy, ethics. His works	980–1037 AD	Persia, Central Asia	Philosophy of Avicenna, his view on Logic, Empiricism and Metaphysics. His works
Waldron HA. (1989)	Textbook	Eng	History, developments of occupational health practice	1700s	Worldwide, Russia	The role of F. Erisman in the development of occupational hygiene and health
Abu Ali ibn Sina (Avicenna) 1979	Book	Russian	Anatomy of human body, philosophy of well-being of a man, causes of disease, manifestations, treatment, dietetics, hygiene, environment al factors etc	980–1037 AD	Persia, Central Asia	Role of environmental factors, including working conditions. Metal poisonings, their treatment
Abu Ali ibn Sina (Avicenna) (1980; 1982)	Book	Russian	Anatomy of human body, philosophy of well-being of a man, causes of disease, manifestations, treatment, dietetics, hygiene, environment al factors etc	980–1037 AD	Persia, Central Asia	Role of environmental factors, including working conditions Metal poisonings, their treatment
Kosarev VV, Babanov SA., (2010)	Textbook	Russian	History, etiology, manifestations, diagnostics, treatment and prevention main of occupational diseases	1760–1780	Russia	Ventilation and fastening of mining workings by M.V. Lomonosov
Izmerov NF. (2011)	National guideline	Russian	History, etiology, manifestations, diagnostics, treatment and prevention main of occupational diseases	1700s	Russia	Focus on workers’ health protection in Russia in 18^th^ century

### History of education in OHS in former soviet republics

Since ancient times, the training of medical doctors has carried out in Central Asia, mainly at the Zoroastrian temples. Along with traditional medicine, doctors who did not receive special medical training used conspiracies and other mystical devices which had significant influence. In Central Asian cities, secular medical schools existed in the first half of the 1^st^ millennium AD and already prepared medical professionals. In the cities, secular doctors had free medical practice. Nestorian doctors who fled from Byzantium, and Indian Buddhist doctors were among those doctors [3].

In Central Asia, like in ancient Iran, the elements of the specialization of medical activities were noted: there were surgeons and special physicians engaged in cerebrovascular and neurological diseases management, eye diseases, dental diseases, mental illnesses and assisted in childbirth [4,5,6,7,8,9,10]. No later than 5–6 centuries later, there were facilities for inpatient treatment of patients and charity of the disabled at temples and in cities [11,12,13,14,15].

They paid great attention to hygienic measures: cleanliness of the body, housing and clothing, supervision of domestic animals, control of insects and rodents, diet and sexuality, hygiene of a pregnant woman and a nursing mother [4,7,16]. Particular attention was paid to working conditions and the prevention of occupational poisoning [17]. Thus Abu Ali Hussein ibn Abdallah Ibn Sino, better known as Avicenna (980–1037), in his famous “Canon of Medical Science”, described the causes and clinical manifestations and treatment of lead poisoning, mercury and other salts [10,11,17,18]. In his “Treatise on Hygiene” he describes the great importance of environmental factors, including the workplace conditions [19,20]. The latter development of labor protection and occupational health is closely linked with the development of workers’ health protection in Russia.

In Russia, the development of the science of labor hygiene and occupational diseases is associated with the development and growth of industry. The history of supervision of industrial production has dates back to the reign of Peter I. In 1719, Peter I approved the Decree on the establishment of the Berg-Collegium, to monitor the mining industry. In 1734, the Empress Anna issued a decree on labor protection and surveillance of working conditions, for which she employed 4 commissars to “deal with for the best for the factories”. In 1744, a law was issued that regulated work in factories and plants and limited night shift duration [21,22].

The workers’ health protection in Russia was reflected in the works of M.V. Lomonosov, A.N. Nikitin, D.P. Nikolsky and others [23,24]. In 1763, in the treatise “The First Foundations of Metallurgy or Ore Mining”, M.V. Lomonosov spoke in favor of the necessity of ventilation of mines, fastening of mine workings, removal of mine waters, protective clothes for miners, etc. In 1841, Russia issued the first law on the work-norm setting – “Work regulations for cloth and ship factories [25].”

The first Russian book on occupational diseases is the “Diseases of Workers with Indications of Protective Measures” (1847) by physician A.N. Nikitin, the first Russian to analyze and describe 120 professions whose representatives develop diseases. He also published in the journal “Friend of Health” a number of articles on protective measures against diseases in various industries [26].

A.P. Dobroslavin (1842–1889), one of the founders of Russian hygiene, described the working conditions at tobacco factories, in mines, caissons, and a clinic of pneumoconiosis of various etiologies, lead and hydrogen sulfide poisoning [25]. His reasoning about the need to study all the factors of the labor process, which can affect the health and work capacity of a person, fully correspond to the scientific concepts of today.

E.M. Dementieva greatly influenced the development of occupational health in Russia in “Factory, what it gives the population and what it takes from it” (1893), in which he painted a vivid picture of the influence of sanitary conditions of labor on the physical development of workers [24]. The mass scale and severity of occupational diseases among workers in that time attracted the attention of public doctors.

At the end of the 19th century, under the guidance of the first hygiene professor at the Faculty of Medicine of the Moscow Imperial University, F.F. Erisman (1842–1915), a group of local sanitary doctors examined 1080 factories and plants in the Moscow province and 114 thousand workers. The doctors published the survey results in 19 volumes (over 6,000 pages), and the results were valuable in characterizing the status of the working class in Russia [26,27,28,29].

Erisman’s “Professional Hygiene, or the Hygiene of Mental and Physical Labor” (1877) is the first original publication in Russia on the incidence of workers in various professional groups [28].

G.V. Khlopin (1863–1929) made a significant contribution in the development of preventive medicine [26,30] through large-scale experimental work on the effect of industrial poisons on the body, the physiology of labor (energy expenditure) and occupational pathology in the chemical and mining industries.

In 1882, Russian officials issued a law concerning the organization of a factory inspection and on the work of minors. Night shift was completely banned for children under 15 years old, and for teenagers aged 12 to 15 years, an eight-hour day was allowed [30].

In 1918, in post-revolutionary Russia, the Commissariat of Labor compiled a list of hazardous industries and professions and subsequently introduced, the issuance of protective clothing in all hazardous industries [24].

The rapid development of the labor protection and inspection system in Russia and in the republics of Central Asia is associated with the emergence of Soviet power. In 1918, the first “Code of Labor Laws” was approved. In 1919, the State Industrial Sanitary Inspection was formed. In 1923, the Moscow Department of Health put forward the slogan “From the fight against epidemics to the sanitation of labor and life.” In 1923, the department established the Research Institute for Occupational Diseases (now the Scientific Research Institute of Occupational Health of the Russian Academy of Medical Sciences) and the first occupational diseases clinic in Moscow [24].

Later in the 1920s, research institutes of labor hygiene and occupational health were established in other parts of Russia and in Ukraine. At first, the course of occupational health at the medical faculties was taught at the departments of therapy. Later, as a result of the expansion of the sanitary and hygiene faculties, the teaching of labor hygiene and occupational health in medical universities began to be conducted in specially organized clinics of occupational health and at the labor hygiene chairs.

The decision of the Central Committee of the VKP (b) of December 18, 1929, “On medical care for workers and peasants”, put the need for preferential servicing of industrial workers in the leading industries as a primary goal of public health. A wide network of health centers at the enterprises were subsequently created in the USSR [25].

With the introduction of medical preventive examinations at industrial enterprises, the study of occupational diseases began to develop taking into account the morbidity and its causes in various industries and the toxicity of substances used in industry. Since 1933, the State Sanitary Inspectorate has started to monitor the performance of existing legislation by industrial enterprises.

In 1939, the first all-Union sanitary norms and rules for the construction design of industrial enterprises were developed and published by the construction and architecture ministry.

In the USSR, in order to prevent exposure to hazardous occupational factors at workplace, workers were given protective clothing, footwear, personal protective equipment, and preventive nutrition at the expense of the employer in accordance with the adopted norms [24].

In the Soviet Union, for the first time in the world, medical and sanitary units were set up and preventive and curative work was performed at enterprises. The creation of sanitary and epidemiological stations on the territorial principle was also completely innovative.

During this period, extremely high rates of industrial development were combined with a decrease in occupational disease incidence.

In the years before the World War II, the issues of the pathogenesis of occupational diseases were studied in the USSR. This helped in the development of issues of diagnosis, treatment, and rational employment of patients with occupational disease. A number of works of Soviet scientists in the field of dust pathology, occupational diseases of blood, etc. have acquired world significance.

During the World War II, a network of medical and sanitary units at enterprises was reinforced in the USSR, the number of which had already increased to 145 in 1942 [24].

In the postwar years, rapid recovery of the national economy, rapid growth of metallurgical, chemical, mining and light industry were due to well-established preventive work on the background of a general decline in occupational pathology. So only in the mining industry for 10 post-war years the incidence of workers has decreased by 13.5 times.

The first research establishment in Central Asia was the Research Institute of Sanitation, Hygiene and Occupational Health (RISHOH) created in Tashkent in 1934 in accordance with the decree of the Council of People’s Commissars of Uzbekistan No. 685 of July 16, 1934.

After World War II, the sanitary and hygiene faculties with departments of labor hygiene and occupational health were established in the Central Asian soviet republics.

In Kyrgyzstan, such faculty with the Department of Hygiene was created at the Kyrgyz State Medical Institute only in 1953. The occupational health course was taught within the Internal Diseases course and a separate course only in 1980.

In 1958, the Ministry of Health of the Kazakh SSR established the National Research Center of Labor Hygiene and Occupational Health (NRCLHOH) in Karaganda [31]. The first Department of labor hygiene in Kazakhstan was established in 1963 at the Karaganda State Medical Institute and of Occupational Health – in 1966.

### The modern state of the study of OHS in Central Asian countries

Currently, the two above mentioned research centers are engaged in scientific research in the field of labor hygiene and occupational health: the RISHOH in Tashkent (Uzbekistan) and the NRCLHOH in Karaganda (Kazakhstan) [31,32,33].

Their main scientific goal is the establishment of patterns in the formation of the industrial environment at industrial enterprises, the hygienic assessment of the occupational risk of workers, the development of measures to optimize working conditions, the prevention of occupational and work-related diseases on the basis of risk theory.

In the field of industrial toxicology, the main task is to determine the extent and nature of the biological effect, based on an assessment of the toxicity and hazard introduced by new chemical compounds.

Another activity of these research centers is the assessment and the certification of workplaces, the development of sanitary and technical passports, as well as in-depth medical examinations of workers of various enterprises and institutions.

The hospitals within these scientific centers handles diagnostics, treatment, medical examinations, expertise of professional fitness and medical rehabilitation of patients with various forms of occupational diseases, as well as prevention of occupational and general diseases in workers exposed to harmful occupational factors.

The occupational health hospital in Uzbekistan has 100 beds, a dispensary department for 33,000 visits per year, three laboratories, a pharmacy, a power unit and other auxiliary departments.

The occupational health hospital in Kazakhstan has four branches for total 117 beds in Karaganda, Ust-Kamenogorsk, Chimkent and Aktyubinsk cities [31].

In Kyrgyzstan, there is an occupational health unit with 10 beds at the National hospital in Bishkek.

In Central Asia, 10 medical universities run the undergraduate and postgraduate programs in Labor Hygiene and Occupational Health: five in Kazakhstan, two in Uzbekistan and one in Kyrgyzstan, Tajikistan and Turkmenistan each.

The summary of the characteristics of courses on Occupational Health, Labor Hygiene, Labor Protection and Safety taught in these countries is presented in the Table 2. The number of credit hours for training on Labor Hygiene in medical universities ranges from 6 to 10.2 credits, on occupational health 1.75 to 3 credits. The purpose of training students in Labor Hygiene is to provide basic theoretical and practical training of a physician in the field of Labor Hygiene and Physiology, which allows physicians to solve current sanitary and epidemiological surveillance issues at facilities under construction and projected facilities for industrial and agricultural purposes, and also to substantiate and develop a set of preventive recommendations and ways for their implementation based on legislation documents. The aim of training medical students on Occupational Health to enable them to timely diagnose occupational and work-related diseases and to organize proper treatment of patients.

**Table 2:** The summary of description of curricula and syllabi in Occupational Health and related subjects in Central Asian countries.

	Kazakhstan	Kyrgyzstan	Tajikistan	Uzbekistan

Curricula (total numbers)				

Medical universities	5	1	1	2
Technical universities	2	2	1	2
Pedagogical universities	1	2	1	1
**Syllabi (total number)**				

Medical universities	5	1	1	1

Features of course syllabi:	**Labor Hygiene**	**Labor Hygiene**	**Labor Hygiene**	**Labor Hygiene**
	Credits: 10.2 Focus areas: – Risk Assessment – OHS in mining, oil industry and agriculture – Certification of working places	Credits: 6 Focus areas: – Labor physiology and OHS in industries in altitude conditions – OHS in agriculture – Certification of working places	Credits: 6.6 Focus areas: – Risk Assessment – OHS in oil & gas industry – OHS in agriculture – Toxicity of new pesticides & chemicals	Credits: 9.5 Focus areas: – OHS in mining, metal industry and agriculture – Toxicology of pesticides
	**Occupational Health**	**Occupational Health**	**Occupational Health**	**Occupational Health**
	Credits: 3 Focus areas: – Medical examination of workers – Rehabilitation of patients OD and work-related diseases	Credits: 1.75 Focus areas: – Lead intoxication – Brucellosis in farmers – Work-related infectious diseases	Credits: 2 Focus areas: – Molecular and sub-cell bases of effects of physical and chemical factors	Credits: 3 Focus areas: – Medical examination of workers – Work-related diseases

Technical universities	2	1	1	1

Features of syllabi:	Total: 7–8 credits Subjects: – Labor Protection by Industry, – Fundamentals of Labor Protection, – Civil Protection, – Industrial Safety	Total: 7–8 credits Subjects: – Labor Protection by Industry, – Fundamentals of Labor Protection, – Civil Protection, – Industrial Safety	Total: 7–8 credits Subjects: – Labor Protection by Industry, – Fundamentals of Labor Protection, – Civil Protection, – Industrial Safety	Total: 8.5–9.4 credits Subjects: – Labor Protection by Industry, – Fundamentals of Labor Protection, – Civil Protection, – Industrial Safety
	CPD training on Occupational Safety 1–2 credits Retraining course for labor protection & safety: 7credits	CPD training on Occupational Safety 1–2 credits Retraining course for labor protection & safety: 7credits	CPD training on Occupational Safety 1–2 credits Retraining course for labor protection & safety: 7credits	CPD training on Occupational Safety 1–2 credits Retraining course for labor protection & safety: 7credits

Pedagogical universities	1	1	1	1

Features:	Life Safety 5 credits	Life Safety 4 credits	Life Safety 4 credits	Life Safety 4 credits

Beside medical universities, the main technical universities of this region prepare specialists for labor protection and safety [32,33,34,35]. In all technical (transport specialists, architects, engineers, geologists, designers, construction technology, food technology, machine technology, IT) and pedagogical universities of all republics, teaching of occupational safety is compulsory. Disciplines such as “Life Safety”, “Fundamentals of Labor Protection”, “Labor Protection by Industry”, “Civil Protection” and “Industrial Safety.” Time allocated for training in occupational health and safety varied from 4 to 9.4 credits. As continuing professional development for all non-medical specialists, training in occupational safety is recommended at 1 credit (basic training), 2 credits (repeated training after 3–5 years of work). For professional retraining of labor protection and safety specialists there is courses of up to 7 credits.

Reviewing the educational programs, we decided to highlight the features of some good examples of training programs from different countries of this region.

### Some examples of good practice from Central Asian countries

#### 1. Kyrgyzstan: Occupational health and safety in conditions of high altitude

The Chair of environmental studies and the Chair of hospital therapy at the Kyrgyz State Medical Academy introduced course of Occupational Health and Safety in High Altitude into their undergraduate program of Preventive Medicine. This course integrated all collective knowledge regarding features of effects of main occupational factors on the background of external hypoxia at industries in high altitude. The main bulk of knowledge bulk is comprised of local researchers from Institute for High Altitude Physiology and Medicine on quantification of changes in energy expenditures, cardiovascular, respiratory systems, thermoregulation, psychomotor status and other systems of human body during labor process at different altitude above sea level. The course participants are to master skills on formulation of recommendation on work-norm setting: duration and organization of daily work and rest, work shift and days-off, compensation rate for salary, etc.

At the Kyrgyz technical university, at all faculties they introduced the subject of Industrial Safety. For students of mining and geology, the department of Industrial Safety introduced new program “Risk management in mining”. The university is actively introducing e-learning with the use of problem based learning.

#### 2. Kazakhstan: Distant and on-site CPD courses in OHS

Due to a need for the training and retraining of specialists, the National Center of Industrial Hygiene and Occupational Health at the Kazakh Ministry of Health and the Chair of Labor Hygiene at the Karaganda State Medical University are main producers of specialists in occupational health and safety in Kazakhstan [34]. They developed eight new distant continuing professional development courses, including “Preliminary and periodical medical examination of workers”, “Occupational health risk assessment in mining”, “Occupational health risk assessment in oil industry”, “Certification of industries on working conditions”, etc [36].

#### 3. Uzbekistan: Occupational health of women working in silk production

Specialists of the Research Institute of Sanitation, Hygiene and Occupational Diseases at the Ministry of Health of the Republic of Uzbekistan (NIISH) studied occupational health of women employed in silkworm breeding, grenadier, silk weaving, knitwear and textile industries. The study results formed the basis of postgraduate Preventive Medicine program at the Tashkent Medical Academy.

#### 4. Regional projects: Tempus and Erasmus Plus project on Central Asian network in Environmental and Occupational Health

Because universities perceive their prestige as closely linked to their master & doctoral programs, the lack of resources and expertise can be covered by the development of an appropriate set of PhD and MSc courses in environmental and occupational health to prepare strongly needed graduates in such disciplines. MSc & PhD programs can strongly benefit from international cooperation and from state-of-the-art teaching technologies.

By inititaive of the International school of medicine (Bishkek, Kyrgyzstan) and the University of Milan the regional educational project “Central Asian Network for Education, Research and Innovation in Environmental and Occupational Health” (CANERIEH) is realized in Kyrgyzstan, Kazakhstan and Tajikistan. The project consortium included six medical universities from these Central Asian countries, including the University of Milan (Italy), the University of Gothenburg (Sweden) and University of Tartu (Estonia). Finanical support from the European Commission TEMPUS program allowed the consortium to reach several significant goals.

First, researchers began building capacity in education and research in these fields. Training was provided for teaching staff and researchers. PhD programs were successfully introduced in participating Central Asian universities. Additionally, centers for education, research & innovation in Occupational & Environmental Health were established in these universities and created anetwork for collaborative activities. PhD students are recruited and started their training and research activities.

The second project «STrengthening Network for EdUcaTiOn, Research and Innovation in Environmental HeALth in Asia»/TUTORIAL 2016–2019 is sponsored by European Commission ERASMUS Plus program become a continuation of the first international project.

The main objective of the TUTORIAL project is to strengthen research capacities in partner countries in areas of public health, promote a sound exchange of information and building capacities between higher education institutions of European Union and Central Asia. This objective will be achieved by tuning existing doctoral studies, developing new MSc programs in relevant areas and implementing blended learning approach. The project will enhance the quality and relevance of higher education across European and Central Asian regions and India in environmental and occupational health and improve their capacity for sustainable international cooperation, thus collaborating at the creating a long lasting excellence international network, to continue its activities after the end of the project period.

The revised and newly introduced MSc & PhD programs will strenghen educational & research capacity of health systems in partner countries.

The project will become a base for preparation of skilled & knowledgable environmental and occupational health specialists for health care systems in Central Asian countries.

Establishing Managed Learning Environment systems at universities and introducing blended learning using the problem based learning in teaching and learning will improve students’ thinking skills, problem solving abilities, and collaborative development of knowledge.

Alltogether, it will enhance capacity and quality of higher education and produced human resources for public health systems in Central Asian countries.

## Conclusions

Education in labor hygiene and occupational health in Central Asia are rooted in medieval period. The development of occupational health services and higher education in this filed is connected to the development in Russia and influenced by soviet period in history of the republics.

The analysis of training programs showed that the education in OHS and preparation of qualified specialists in Central Asiana countries is up-to-date and tailored to local needs in prevention of risks of accidents and injuries and promotion of a preventive culture in the workplace. The universities try to stay in accordance with EU developments and to internationalize education in this filed. Such action is important for improving health and well-being of the population, rapid development of their societies and economics.

The higher education in occupational health and safety in this region is in line with WHO and ILO directives regarding the OHS education for all competent persons.
